# Diagnostic approach and management of patients with headache in Danish chiropractic practice

**DOI:** 10.1186/s12998-026-00652-0

**Published:** 2026-05-29

**Authors:** Jeppe Pihl Skovbjerg, Henrik Hein Lauridsen, Henrik Wulff Christensen, Rikke Krüger Jensen, Kristina Boe Dissing

**Affiliations:** 1 Chiropractic Knowledge Hub, Odense, Denmark; 2 Center for Muscle and Joint Health, Department of Sports Science and Clinical Biomechanics, University of Southern Denmark, Odense, Denmark

**Keywords:** Headache, Chiropractor, Chiropractic management, Headache management, Manual therapy, Non-pharmacological management, ICDH-3, Guidelines

## Abstract

**Background:**

Headache disorders are among the leading causes of disability globally. In primary care, including chiropractic practice, clinicians often serve as the first point of contact for patients seeking evaluation and management of headache. Despite this, limited evidence exists regarding how Danish chiropractors assess, diagnose, and manage headache in routine practice. This study aimed to examine how Danish chiropractors adopt a profession-specific clinical care standard, conduct clinical assessments, apply the ICHD-3 diagnostic criteria, and manage patients presenting with headache.

**Methods:**

A cross-sectional, questionnaire-based study was conducted between May 2022 and August 2022 among Danish chiropractors. The Danish Headache Questionnaire was used covering aspects such as diagnostic knowledge, clinical assessments and management. Descriptive statistics were used to characterise respondents and their clinical practices. A predefined threshold of ≥ 70% was applied to indicate acceptable adherence to the clinical care standard and sufficient familiarity with the ICHD-3 criteria.

**Results:**

A total of 100 chiropractors completed the questionnaire, corresponding to a response rate of 14.4%. Self-reported data indicated that a larger percentage of Danish chiropractors demonstrate adequate knowledge of and adhere well to the clinical care standard. However, a proportion of respondents reported modest familiarity with specific elements of the standard and knowledge of red flags was limited. Overall familiarity with, and use of, the ICHD-3 diagnostic criteria was high. Management approaches largely aligned with guideline-supported strategies, with most chiropractors reporting the use of manual therapy, exercise, and patient education. In contrast, structured monitoring tools - such as headache diaries - were seldom used.

**Conclusion:**

Respondents generally appear to follow the profession-specific clinical care standard, demonstrate good familiarity with ICHD-3 criteria, and deliver guideline-aligned management for patients with headache. These findings suggest that chiropractors may play a meaningful role in primary-care headache management and potentially help reduce the burden on other health-care providers, although the low response rate warrants caution in generalising these findings. Identified gaps in knowledge and practice indicate a need for targeted postgraduate education, enhanced implementation strategies to support consistent use of clinical care standards and guidelines, and policy initiatives that facilitate the integration of chiropractors into interdisciplinary headache care pathways.

**Supplementary Information:**

The online version contains supplementary material available at 10.1186/s12998-026-00652-0.

## Background

Headaches are a leading cause of disability and a major public health concern worldwide [1–4]. The global one-year prevalence of any headache disorder is estimated at 52% (aged 20 to 85 years) [4]. Among specific types, migraine affects approximately 14% of the population and tension-type headache affects about 26% (aged 10 to 75 + years) [2]. In Denmark, headache poses a similarly substantial burden, with a point prevalence of approximately 18% of the general Danish population experiencing migraines or frequent headaches (aged 16 to 75 + years) [5]. Furthermore, headache imposes a substantial burden on the health care system and the individual. In Denmark, around 75% of respondents in a nationwide survey who reported experiencing headaches had consulted their primary care physician due to headache [6] and more than two thirds have used complementary or alternative therapies [6,7]. Despite these high levels of health care use, 28% of people with headache reported being unable to manage their headache attacks, and 82% reported that headache was a daily burden [6].

Clinical practice guidelines for the management of headache recommend incorporating non-pharmacological interventions, such as patient education, physical activity and manual therapy, as either adjuncts or alternatives to pharmacological treatment [8–11].

Despite established clinical guidelines, headache management in practice does not always adhere to these recommendations. For instance, up to 10% of patients use opioids for pain relief, which is not supported by current guidelines and is associated with a risk of adverse events [12, 13]. Furthermore, a Danish study found that general practitioners referred nearly half of their patients with headaches to neurologists and ordered MRI or CT scans for about one in four, rates the authors deemed unnecessary high [14]. Variability in therapeutic approaches to headache care may compromise both quality of services and patient safety, and current utilization patterns suggest suboptimal management [6]. Following established clinical guidelines is essential to ensure uniform and evidence-based approaches to headache management across providers.

Primary care is the first point of contact for the majority of patients with headaches, and timely and efficient management of headaches in primary care may reduce costly and unnecessary examinations and referrals to specialists in secondary care [15–17]. Danish chiropractors are an integrated part of the primary care system and provide care for patients presenting with headache. A recent study found that 12% of chiropractic patient consultations were related to headache complaints [18]. To promote consistent and evidence-based headache management among chiropractors, the Danish Society of Chiropractic developed a profession-specific clinical care standard for managing patients with headache in 2019 [19]. This care standard recommends using the International Classification of Headache Disorders (ICHD-3) criteria for diagnosing primary and secondary recurrent headaches [20]. It also provides guidance on medical history, physical examination, and management of patients with headache.

However, little is known about how Danish chiropractors implement the recommendations of the care standard in everyday practice. In particular, there is limited information regarding chiropractors’ knowledge of and use of the care standard, their approach to clinical assessment, familiarity with the ICHD-3 diagnostic criteria, and overall management approaches for patients with headaches. Without such insight, it is difficult to evaluate the extent to which the care standard has been translated into routine clinical care and to identify potential areas for improvement.

To address this gap, the aim of the present study was to investigate how Danish chiropractors apply the profession-specific clinical care standard, conduct clinical assessments, use the ICHD-3 diagnostic criteria, and manage patients presenting with headache.

## Methods

The reporting of this study conforms to the Strengthening the Reporting of Observational studies in Epidemiology (STROBE) statement for reporting of observational studies [21].

### Setting and study design

The study was a survey-based cross-sectional study conducted in 2022 among Danish chiropractors working in private practice. Chiropractors in Denmark, work as an integrated part of the primary healthcare system and are regulated by the National Health Authority. The cost of chiropractic treatment is partially covered by the Danish health insurance system, and patients do not require a referral from their general practitioner to access chiropractic services. The 2019 profession-specific clinical care standard for managing patients with headache, hereafter referred to as the care standard, was disseminated to all Danish chiropractors via postal distribution as a pamphlet and made accessible online through the website of the Danish Society of Chiropractic. Furthermore, it was supported by information in newsletters from the Danish Society of Chiropractic, in collaboration with the Danish Chiropractic Association.

### Participants and recruitment

Danish chiropractors working in private practice, who were members of the Danish Chiropractic Association (DCA) as of March 2022, were invited to participate in the study. Since the DCA maintains an almost complete registry of licensed chiropractors, this approach provided a pragmatic means of reaching the target population. Participation involved completing a survey and contributing to data collection of patients with headache presenting to chiropractic practice. Recruitment was conducted via personal emails, and reminder emails were sent one and two weeks following the initial distribution of the survey. Chiropractors who agreed to participate provided written consent and were asked to provide information about their current work situation. Only chiropractors working in primary care clinical practice within private settings were included. Those not engaged in such practice (e.g. researchers, chiropractors working in public hospitals, or those holding university positions) were identified if they selected the response option ‘do not see patients in primary care’. To increase awareness of the project, it was promoted through newsletters, social media platforms, conferences, webinars, and personal contacts. Additional information regarding the project, was made available through webinars and written materials, and on the project website [22].

### Data collection and variables

Data were collected between May 2022 and August 2022 using the Danish Headache Questionnaire (DHQ), administered as an online, questionnaire-based survey. The development and content of the DHQ have been described previously and demonstrated good feasibility and content validity [23].

Data collection was conducted using the Research Electronic Data Capture system (REDCap) hosted at OPEN (Open Patient data Explorative Network, Odense University Hospital, Region of Southern Denmark) [24–26].

The survey included variables on chiropractor characteristics (sex, age, number of days working in clinic per week, years working in clinic, country of education and educational background) and clinic characteristics (clinic location by region of Denmark, number of chiropractors working in the clinic, and other health care professionals working in the clinic). The chiropractors were also asked about their knowledge of the content and application of the Danish care standard [19], including whether using it had influenced their clinical practice.

In addition, the survey explored their familiarity with and use of the diagnostic criteria for diagnosing headaches outlined in the International Classification of Headache Disorders (ICHD-3), covering both primary and secondary headaches. The specific headache types addressed included migraine, tension-type headache, cluster headache, cervicogenic headache, and medication overuse headache. Furthermore, chiropractors reported on routine clinical practice, including history-taking components, physical examination procedures, use of X-ray imaging, awareness of red flags, and familiarity with indications warranting further diagnostic investigation. They also described their approaches to patient management: the types of treatment modalities they provide, how they monitor progress over time, and any side effects observed in response to these treatments. Management practices and use of x-ray were assessed only for migraine, tension-type headache, and cervicogenic headache, as these conditions are the most commonly encountered headache types in chiropractic practice [27–30]. The complete questionnaire is available online through a repository of commonly used instruments [31].

### Statistical analysis

Descriptive statistics were used to characterise the study participants. Continuous variables were summarized as median (IQR) for non-normal distributions and mean (SD) for approximately normal distributions), while categorical variables were presented as frequencies and percentages. Results were displayed in tables or illustrated graphically, as appropriate.

We established a threshold of 70% adherence as an acceptable benchmark for implementing the care standard and for level of familiarity with the diagnostic criteria where appropriate. Following an initial review of the data, the threshold was established post hoc in order to present the results in a clearer and more interpretable manner. This cut-off is a pragmatic indicator of moderate-to-good uptake in clinical guidelines that has previously been used [32]. Also, the most frequently used treatment modalities were defined as those falling within the upper quartile of responses marked ‘Always’ or ‘Often’.

Statistical analyses were conducted using STATA version 18 (StataCorp, College Station, TX, USA). Graphs and other visual representations of the data were generated using Microsoft Excel (Microsoft Corporation, Redmond, WA, USA).

## Results

### Chiropractor characteristics

**Fig. 1:**
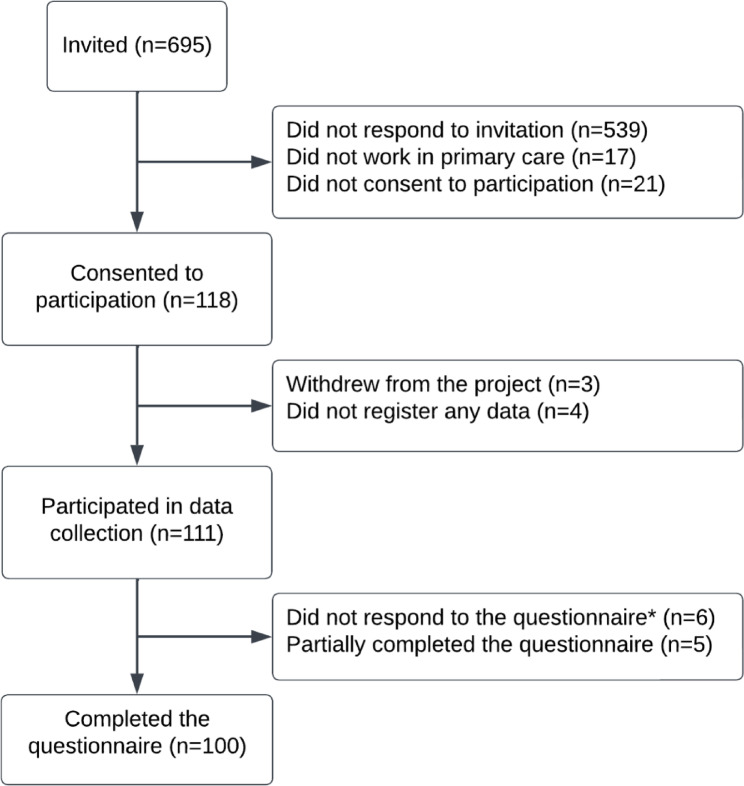
Flowchart of participant recruitment. *Only responded to the questions about clinic data

A total of 695 chiropractors were invited to participate in the study. Of these, 111 (16.0%) consented to participate, and 100 completed the questionnaire and were included in the final analyses, resulting in an overall response rate of 14.4% (95% CI: 11.9–17.2%) (Fig. 1).

The mean age of the participating chiropractors was 41.2 years (SD = 12.6), 62% were female, and 45% had more than 15 years of professional experience (Table 1). Nearly four out of five chiropractors obtained their degree in Denmark and one in five had an additional educational background besides chiropractic training. Almost all chiropractors (98%) worked four or five days per week in the clinic. Chiropractors from four out of five regions of Denmark were well represented, except for the North Denmark region, which included only one participant. Almost 90% of the chiropractors were working in clinics with other chiropractors (median number per clinic: 4 (IQR 3-6.5)), and approximately three quarters of the chiropractors worked in multidisciplinary clinics (*n* = 73). The most common other health care professionals working in the clinics were physiotherapists and massage therapists (Table 1).

**Table 1:** Characteristics of the participating chiropractors

	Total (*N* = 100)
Age in years, mean (SD)*	41.2 (12.6)
Female, n (%)	62 (62%)
Number of days working in clinic per week, n (%)	
1	0 (0%)
2	1 (1%)
3	1 (1%)
4	30 (30%)
5	68 (68%)
Years working in clinic, n (%)	
Internship	10 (10%)
1–4 years	23 (23%)
5–10 years	13 (13%)
11–15 years	9 (9%)
> 15 years	45 (45%)
Country of graduation, n (%)	
Denmark	79 (79%)
England	12 (12%)
USA	8 (8%)
Canada	1 (1%)
Other educational background besides chiropractor, n (%) **	21 (21%)
Clinic location by region of Denmark, n (%)	
The North Denmark Region	1 (1%)
Central Denmark Region	22 (22%)
The Region of Southern Denmark	36 (36%)
Region Zealand	21 (21%)
The Capital Region of Denmark	20 (20%)
Number of chiropractors in the clinic (including the respondent), median (IQR)	4 (3-6.5)
Other health care professionals working in the clinic, n (%)	73 (73%)
General practitioner	1 (1%)
Physiotherapist	57 (57%)
Massage therapist	54 (54%)
Acupuncturist	10 (10%)
Others***	14 (14%)

*There were two missing answers for age

**Bachelor of Sports Science, medical degree, massage therapist, PhD, yoga teacher, shipping degree, economics degree, graphic designer, master’s degree in health information, Bachelor of Mathematics

***Reflexologist, Cranio-sacral therapist, Nutritionist, Psychomotor therapist, Psychotherapist, Occupational therapist, Coach, Body SDS, chiropodist, acupuncturist, Psychologist, Educator of relaxation

SD: standard deviation. IQR: interquartile range

### Knowledge and use of the Danish profession-specific clinical care standard

Almost 60% of the chiropractors reported being familiar with most or all of the content in the care standard, while approximately one-third knew some parts of it, and a small proportion were not familiar with the content at all (Table 2). 42% indicated that their clinical practice was already in compliance with the care standard, while 44% reported that the care standard had led to changes in some aspects of their daily practice. The areas of medical history, physical examination and diagnostic approach were those in which most chiropractors reported changing their daily practice after the care standard was published (Table 2).

**Table 2:** Chiropractors’ knowledge of the Danish profession-specific clinical care standard and its influence on daily practice

Are you familiar with the content of the Danish clinical care standard? *n* (missing)	100 (0)
Yes, all of it, n (%)	9 (9.0)
Yes, the most of it, n (%)	50 (50.0)
Yes, some parts of it, n (%)	37 (37.0)
No, n (%)	4 (4.0)
Has the care standard changed your way of practicing? N (missing)	100 (1)
Yes, some parts of it, n (%)	44 (44.0)
No, my practice was already in line with the care standard, n (%)	42 (42.0)
No, not at all, n (%)	13 (13.0)
If yes, what part of your practice has changed? * N	44
The medical history, n (%)	28 (66.7)
Physical examination, n (%)	25 (65.8)
Diagnostic approach, n (%)	24 (63.2)
Management, n (%)	17 (42.5)
Referrals**, n (%)	14 (38.9)
Other, n (%)	2 (6.9)

* Multiple answers possible. ** Referrals to other health care professionals

### Clinical assessment

#### The self-rated content of history taking and physical examination

More than 70% of the chiropractors reported that they ‘Always’ or ‘Frequently’ collected patient information in accordance with the care standard regarding medical history for all items, ranging from 75% to 100%. The item most frequently assessed was general medical history, reported by all respondents (100%), whereas the item assessed least, was inquiry into general health between headache episodes (74.8%) (Additional File 1, Figure S1).

In relation to the recommended components of the physical examination, two items fell below the 70% threshold for being reported as performed ‘Always’ or ‘Frequently’: walking patterns (28%) and coordination and balance (59%) (Additional File 1, Figure S1). All other items were reported as being performed ‘Always’ or ‘Frequently’ by 75% to 100% of chiropractors.

#### The self-rated use of radiographic imaging (x-ray)

The self-rated use of x-ray met the 70% threshold for being reported as ‘Never’ or ‘Rarely’ used in cases of tension-type headache (81%) and migraine (85%). This was not reached for cervicogenic headache, where 62% reported infrequent use (Additional File 1, Figure S2).

#### Awareness and identification of red flags

Awareness of red flags warranting further (acute) investigation in patients with headache was assessed. Only two items surpassed the 70% threshold: ‘Headache accompanied by fever or neurological symptoms’ (73%) and ‘Thunderclap headache’ (89%). The remaining items showed lower levels of familiarity, ranging from 39% to 68% for the ‘Very familiar’ response option (Additional File 1, Figure S3). The items of least familiarity were ‘Nocturnal headaches in children’ (39%) and ‘New-onset headaches in patients with prior cancer or HIV’ (39%).

### Familiarity with the ICHD-3 diagnostic criteria

The majority of chiropractors reported using the ICHD-3 diagnostic criteria for identifying both primary (92%) and secondary headaches (85%). For primary headaches, 65% found the criteria helpful for communication with other health care professionals, 74% believed the criteria impacted management, 65% considered them applicable for the patients, 74% found them easy to use, and 86% found the criteria clear. The corresponding figures for secondary headaches were comparable (Additional File 1, Figure S4 and S5.

**Fig. 2:**
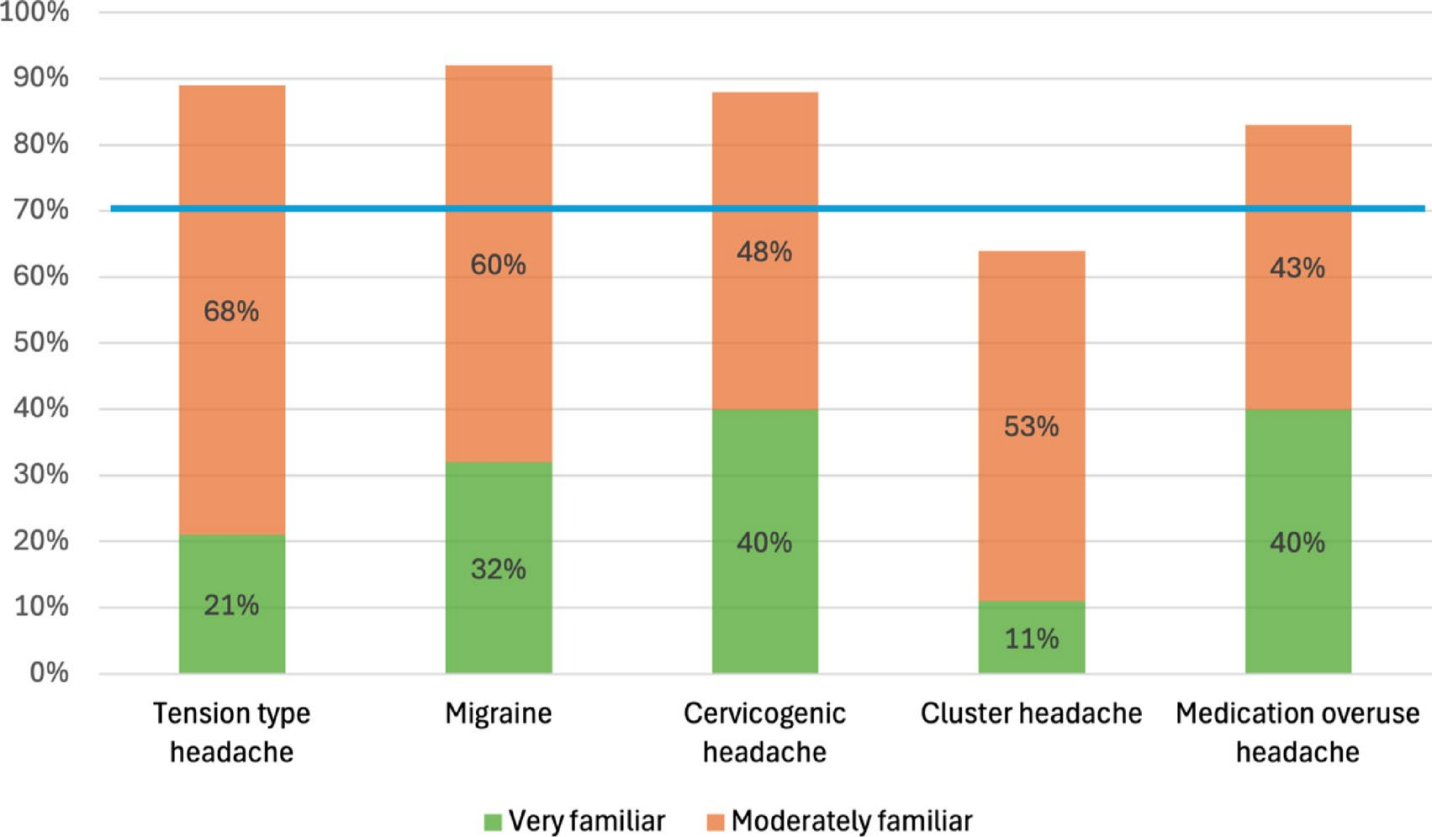
Chiropractors’ self-rated knowledge of diagnostic criteria for diagnosing headaches (ICHD-3). *Note*. Blue line: 70% threshold for adherence

Chiropractors reported varying levels of familiarity with the diagnostic criteria. The predefined threshold of 70% was met for most headache types explored (ranging from 83% to 92%), except for cluster headache (64%) (Fig. 2). 9% of the respondents reported that they were not familiar with the criteria (Additional File 2, Table S1).

Across the specific items within the diagnostic criteria, chiropractors were generally ‘Very familiar’ with most of the content (Additional File 1, Figures S6-S8), with familiarity levels ranging from 56% to 96% depending on the headache type. Regarding tension-type headache, four items did not reach the predetermined threshold of 70%: number of episodes of headache for all three types (sporadic, episodic and chronic) and duration of episodic tension-type headache (Additional File 1, Figure S6). For cervicogenic headache, the two items within the diagnostic criteria that did not reach the 70% threshold were ‘Clinical or radiological evidence of disease or lesion of the cervical spine’ and ‘The headache disappears as a result of a diagnostic blockade of the cervical spine’ (Additional File 1, Figure S7). For migraine, the familiarity level was above 70% for all items (Additional File 1, Figure S8).

### Management approaches

#### Treatment modalities

Across the three types of headaches explored, ‘Manipulation’ and ‘Soft tissue treatment’ emerged as the most frequently used treatment modalities. For tension type headache, both were reported by 97% of the chiropractors; for migraine 83% and 78%, and for cervicogenic headache 98% and 92%, respectively (Additional File 2, Table S2). Additionally, more than 80% of chiropractors reported using exercise to manage tension-type and cervicogenic headache. The use of advice on stress management, dietary habits, physical activity, and headache triggers varied widely across headache types (ranging from 12% to 81%). A full list of all modalities reported is provided in Additional File 2, Table S2.

#### Use of monitoring tools

The chiropractors did not routinely use a headache diary or calendar for diagnosing or monitoring their patients’ headaches and did not meet the threshold of 70% (Additional File 2, Table S3). Only a small proportion of chiropractors reported frequent use of a headache diary or calendar, with 7% and 5% indicating that they used these tools ‘Always’ or ‘Often’, respectively (Additional File 2, Table S3).

#### Side-effects experienced

The most common side effects noted by chiropractors after treatment were “Provocation of known headache and symptoms” (38%), “Soreness” (25%) and “Tiredness” (20%) (Additional File 2, Table S4). No serious side effects were reported.

## Discussion

Based on self-reported data, Danish chiropractors generally indicated adequate knowledge of and good adherence to the profession-specific clinical care standard, incorporating it into clinical assessments, although some reported unfamiliarity with certain parts of its content. Familiarity with the ICHD-3 diagnostic criteria was generally high. Regarding management, most followed guideline-based approaches including manual therapy, exercise and patient education; however, monitoring tools were rarely utilised.

### Knowledge and use of the Danish profession specific clinical care standard

That nearly 40% of chiropractors are unfamiliar with parts of the care standard’s content raises concerns about the consistency and quality of care provided. Such gaps may contribute to variability in clinical practice potentially undermining the standardization efforts the care standard aims to promote. It suggests that implementation strategies may have been insufficient within the profession. Besides distributing the care standard to all chiropractors, there was no follow-up on how to implement it or on whether it had been implemented or used. However, it is noteworthy that 44% of participants reported that the care standard had changed their practice, while 42% indicated they were already in compliance. This near-even split suggests two complementary interpretations. On one hand, the care standard may have been highly effective at influencing clinician behaviour. On the other hand, the findings may indicate that many clinicians were already providing care aligned with best-practice recommendations, and the standard primarily served to validate existing high-quality practice. Even so, some scope for improvements remains. Implementation efforts could have included supportive strategies, such as webinars or workshops using case-based examples to demonstrate how the care standard could be applied in clinical practice. In addition, follow-up evaluation through practitioner surveys, audits, or feedback mechanisms could have helped assess awareness, uptake, and barriers to implementation.

### Clinical assessment

#### The self-rated content of history taking and physical examination

To our knowledge, no primary-care studies have evaluated the content of history taking and physical examination in headache consultations. In our sample, adherence to recommended items in the medical history was generally high. However, the item concerning general health between episodes had the lowest adherence, despite its potential relevance for tailoring care. For example, if new comorbidities affect the patient’s ability to self-manage or engage in exercise, or if newly prescribed medications influence their condition, the management plan may need to be adjusted accordingly. The physical examination adherence was generally good, but assessments of walking patterns and coordination/balance fell below the desired threshold - elements important for identifying possible serious secondary underlying conditions.

These patterns raise questions about clinical prioritization, such as whether all assessment items perceived as equally valuable in diagnostic reasoning and what drives the omission of certain components. Clarifying this is crucial for ensuring that all relevant aspects of patient evaluation are consistently applied, thereby supporting accurate diagnoses and optimal care planning. Notably, a thorough history often suffices to exclude serious secondary causes of headache, reducing the need for a more comprehensive examination [11, 20].

#### The self-rated use of radiographic imaging (x-ray)

X-ray was used infrequently as a diagnostic test by most. When obtained, it was mainly to support a suspected diagnosis of cervicogenic headache or rule out underlying pathology. In general, x-ray is not recommended as a diagnostic tool when diagnosing headache [33, 34], nor is it endorsed by the Danish profession-specific clinical care standard [19]. However, one item in the ICHD-3 diagnostic criteria of cervicogenic headache includes ‘Clinical or radiological evidence of disease or lesion of the cervical spine’ [20], which may partly explain why some chiropractors use x-ray. This should be of concern when developing future guidelines and be aligned with the ‘Choose Wisely’ campaign for reducing unnecessary examinations and procedures [35]. There has been debate regarding the diagnosis of cervicogenic headache [36, 37], and an alternative set of diagnostic criteria exists, which does not incorporate imaging and may be more clinically useful (the Cervicogenic Headache International Study Group (CHISG) criteria) [38]. Nevertheless, we chose to use the ICHD-3 criteria, as they remain the widely accepted standard for headache diagnosis and are employed in the majority of related studies.

#### Awareness and identification of red flags

In general, familiarity with red flags as indicators for acute investigation was not high, only two items reached the threshold: ‘Headache accompanied by neurological symptoms’ (73%) and ‘Thunderclap headache’ (89%). This suggests that while the most critical and widely recognized warning signs are relatively well understood, awareness of other red flags may be insufficient. Such gaps could lead to delayed recognition of serious underlying conditions, such as cerebral haemorrhage, brain tumours or meningitis, highlighting the need for targeted education and clearer clinical guidance to improve early detection and referral practices.

### Familiarity with the ICHD-3 diagnostic criteria

Evidence on primary care clinicians’ familiarity with, and use of, the diagnostic criteria for headaches is limited and focuses mainly on general practitioners [28, 39]. In Norway, only 5% used ICHD-3 criteria in more than half of their cases and 50% found headache as a challenging problem [40]. In the USA, 35% felt very comfortable diagnosing migraine [41], while in the UK 70% of the patients were not given a diagnosis by the general practitioners [42]. These observations underpin the findings of a Danish study highlighting the need for more general practitioner education in diagnosing and managing headaches [14, 43].

For chiropractors, an Australian study [44] reported high familiarity with the criteria for diagnosing primary and secondary headaches (98% and 81%) and actively using these criteria in practice (85% and 90%). This is in line with our study, where the participating chiropractors reported that their diagnosis of primary (92%) and secondary headaches (85%) were largely based on the diagnostic criteria (ICHD-3).

Differences between general practitioners and chiropractors may be attributable to variation in both consultation structure and scope of practice. General practitioners typically operate under substantial time constraints due to high patient volumes [45] and the need to address a broad spectrum of acute and chronic conditions. In contrast, Danish chiropractors generally schedule longer appointments [46] and concentrate exclusively on musculoskeletal disorders, including certain headache types. These differences in consultation length and clinical focus likely contribute to the observed variation in how general practitioners and chiropractors diagnose patients presenting with headache.

The perceived applicability of the diagnostic criteria varied: in our study, 65% to 86% of respondents expressed positive views (Additional File 1, Figure S1 and S2), whereas the Australian study reported a range of 48% to 86%. Thus, a substantial proportion in both settings do not consider the criteria fully applicable. Consequently, when diagnostic criteria are viewed as impractical, clinicians may rely on subjective judgment or alternative approaches, leading to inconsistent diagnoses, variability in patient management, and reduced opportunities for evidence-based care.

Familiarity with the specific items within the diagnostic criteria for migraine, tension-type headache, and cervicogenic headache generally met the 70% threshold as an indicator for acceptable familiarity for most items. However, familiarity was lowest for tension-type headache - particularly for frequency and duration - which did not reach the threshold. This contrasts with a recent Danish study, which found that tension-type headache is the most commonly seen headache in Danish chiropractic practice [47], suggesting that diagnoses may not consistently follow the diagnostic criteria or that overlapping features make differentiation challenging.

### Management approaches

#### Treatment modalities

Guidelines recommend non-pharmacological treatment when managing hedache [9–11, 19], either as a stand-alone or an adjunct treatment, to standard care, tailored to patient preferences. In this study, spinal manipulation and myofascial treatment modalities were the most frequently selected across headache types, followed by exercise and, less often, advice on active living. Exercise was selected less often for migraine compared to tension type headache and cervicogenic headache, likely because activity may aggravate migraine during attacks, despite having a positive effect on reducing both frequency and intensity of migraine [48, 49]. Advice on active living was mainly reported for tension-type headache, consistent with its links to modifiable factors such as muscle tension and stress. Overall respondents appeared to combine modalities as recommended by clinical practice guidelines [9, 11].

Our findings differed from those of the Australian study [44], where headache triggers and stress management were the most common treatments for migraine and tension-type headache, followed by soft tissue and manipulative therapies. For cervicogenic headache, manipulative therapy and exercises predominated. Such differences between countries may reflect differences in local practice contexts, healthcare systems, or patient expectations.

#### Use of monitoring tools

Headache diaries and calendars are recommended for diagnosis and management of headache [15, 19]. In our study, frequent use was uncommon (< 7%) which contrasts with higher rates observed among Australian chiropractors (16%) [44] and general practitioners (33% to 35%) [14, 40]. A likely explanation includes differences in professional roles and clinical workflows which may influence the adoption of monitoring tools. Second, perceived utility and practicality could also play a role, as some clinicians may consider diaries time-consuming or question their added value compared to patient interviews. Third, variations in training and guideline emphasis across professions may affect awareness and confidence in using such tools. Fourth, patient characteristics and headache severity in the populations treated could influence whether diaries are deemed necessary. Finally, low uptake of monitoring tools may also reflect uncertainty about how to interpret diary data and integrate it into routine care, as well as implementation barriers. While Danish general practitioners can access headache monitoring tools directly within electronic health records, these tools are not currently integrated into chiropractic electronic systems and must be documented separately. This added administrative burden is time-consuming and may be deprioritised in busy practice.

#### Side-effects experienced

No severe side-effects were reported in response to treatments. Side effects were typically benign and transient, and included provocation of known headache, soreness, and tiredness. This is consistent with other studies examining adverse events following manual treatment [50, 51], suggesting that non-pharmacological treatment is generally safe.

### Strength and limitations

All members of the Danish Chiropractic Association were invited to participate in the study. This invitation was supplemented by personal contacts to increase the participation rate. However, participant distribution by region, gender, and age differed from the demographic profile of Danish chiropractors, limiting representativeness [52]. Particularly the North Denmark Region was represented by only one participant. The low response rate of 14% may reflect a selection bias, either because chiropractors with a particular interest in headache care were more motivated to participate, or because others were unable or unwilling to allocate the time required for data collection. Nevertheless, this may have influenced our results if the participating chiropractors were more motivated or had a specific professional interest in headache management than the non-participants. Such self-selection could lead to overestimated adherence, as clinicians with greater engagement in the topic may be more likely to follow recommended practices. Consequently, the observed level of clinical adherence should be interpreted as an *upper bound* - that is, a best-case estimate - rather than a reflection of routine practice across the broader Danish chiropractic population, where adherence is likely to be lower.

However, we have no information about the non-participants and are therefore unable to determine whether they differ in any way from the participants. While low response is common in surveys targeting health care professionals in private practice [53, 54], higher rates have been achieved elsewhere (Australia 36% [44], France 46% [55]), suggesting recruitment strategies and professional engagement may influence participation.

Asking questions about both knowledge of the individual diagnostic criteria and overall knowledge provides insights into specific knowledge gaps. However, the diagnostic scope was limited, as not all ICHD-3 subclassifications (e.g., chronic vs. non-chronic) were covered. This can, potentially, impact findings on knowledge and management of different headache types.

The study provides a comprehensive description of chiropractors’ management of headache patients, covering knowledge of ICHD-3 criteria, red flags, national care standards, interdisciplinary management, and treatment modalities. Nevertheless, self-reported data are vulnerable to social desirability bias, in which respondents overstate socially approved knowledge or behaviours and downplay less desirable ones, potentially inflating estimates of competence and adherence [56]. This response style is well documented in survey research and may distort observed associations in self-report studies. Recall bias is a potential concern, as participants may not accurately remember past events or may be reluctant to report certain information. For example, when reporting side effects, clinicians may recall primarily the more severe events but may hesitate to report them due to feelings of shame or concern about perceived competence. Reporting bias was also apparent in our findings: while adherence to taking medical histories was reported as high, the use of objective monitoring tools, such as headache diaries, was comparatively lower. This discrepancy suggests that, although clinicians are aware of the recommended practices for patient assessment, the consistent use of structured tools in practice may be limited.

Finally, the 70% adherence threshold was chosen pragmatically, acknowledging its arbitrary nature. In exploratory and implementation research, benchmarks are often selected based on feasibility rather than theoretical justification. For example, studies employing expert consensus methods, such as Delphi panels, commonly use agreement thresholds of 70–80%, reflecting a balance between rigor and practicality [57]. Similarly, Burgers et al. have defined high compliance rate as ranging from 70% to 100% [32]. Given the absence of agreed definitions and standardised measures of adherence, this indicates the complexity of assessing this construct in clinical practice.

## Conclusion

This study suggests that, among respondents, Danish chiropractors generally adhere to the profession-specific clinical care standard and are familiar with the ICHD-3 diagnostic criteria, with management practices largely consistent with guideline recommendations, emphasizing manual therapy, exercise, and patient education. However, due to the low response rate, the results may reflect an upper bound, likely overestimating adherence within the chiropractic profession, and may therefore not be representative of the Danish chiropractors more broadly. The findings of this study indicates that Danish chiropractors can play an important role in headache management within primary care and may contribute to reducing the burden on other health care providers. Nonetheless, identified gaps in knowledge, practice and awareness of red flags underscore the need for targeted post-graduate education, systematic implementation strategies of clinical care standards and guidelines, and policy initiatives that support integration of chiropractors into interdisciplinary headache care pathways.

## Supplementary Information

Below is the link to the electronic supplementary material.

Supplementary Material 1

Supplementary Material 2

Supplementary Material 3

## Data Availability

The datasets used and analysed during the current study are available from the corresponding author on reasonable request.

## Abbreviations

DCA: Danish chiropractic association
DHQ: The Danish headache questionnaire
ICHD-3: International classification of headache disorders version 3
IQR: Interquartile range
REDCap: Research electronic data capture system
SD: Standard deviation
STROBE: Strengthening the reporting of observational studies in epidemiology
