# Supplementary material for: Diagnostic approach and management of patients with headache in Danish chiropractic practice
Source: Chiropr Man Therap. 2026 May 29;34:29. doi: 10.1186/s12998-026-00652-0 (PMC13419007; doi:10.1186/s12998-026-00652-0)
Supplement: Supplementary file 1 — Supplementary Material 1 [file 12998_2026_652_MOESM1_ESM.docx]

# Additional File 2: Supplemental Tables

Table S1 Chiropractors’ self-rated knowledge of diagnostic criteria for diagnosing headaches (ICHD3)

| N=100 | Very familiar | Moderately familiar | Slightly familiar | Not familiar |
| --- | --- | --- | --- | --- |
| Tension type headache, n (%) | 21 (21.0) | 68 (68.0) | 11 (11.0) | 0 (0.0) |
| Migraine, n (%) | 32 (32.0) | 60 (60.0) | 7 (7.0) | 1 (1.0) |
| Cervicogenic headache, n (%) | 40 (40.0) | 48 (48.0) | 11 (11.0) | 1 (1.0) |
| Cluster headache, n (%) | 11 (11.0) | 53 (53.0) | 29 (29.0) | 5 (5.0) |
| Medicine overuse headache, n (%) | 40 (40.0) | 43 (43.0) | 14 (14.0) | 2 (2.0) |

Table S2 Treatment modalities used in management of patients with headache*

|  | Tension type headache, n (%) | Missing, n (%) | Migraine, n (%) | Missing, n (%) | Cervicogenic, n (%) | Missing, n (%) |
| --- | --- | --- | --- | --- | --- | --- |
| Manipulation | **97 (97.0)** | 0 (0.0) | **82 (82.9)** | 1 (1.0) | **98 (98.0)** | 0 (0.0) |
| Mobilization without impulse | 34 (35.8) | 5 (5.0) | 30 (30.9 | 3 (3.0) | 35 (35.7) | 2 (2.0) |
| Activator | 11 (11.0) | 1 (1.0) | 12 (12.2) | 2 (2.0) | 9 (9.1) | 1 (1.0) |
| Drop treatment | 18 (18.0) | 0 (0.0) | 19 (19.2) | 1 (1.0) | 18 (18.3) | 2 (2.0) |
| Toggle recoil | 8 (8.2) | 2 (2.0) | 7 (7.2) | 3 (3.0) | 7 (7.2) | 3 (3.0) |
| Treatment of the temporomandibular joint | 20 (20.0) | 0 (0.0) | 12 (12.1) | 1 (1.0) | 14 (14.0) | 0 (0.0) |
| Massage, myofascial technique and trigger points | **97 (97.0)** | 0 (0.0) | **78 (78.0)** | 1 (1.0) | **92 (92.0)** | 0 (0.0) |
| Dry needling/acupuncture | 44 (44.4) | 1 (1.0) | 34 (34.7) | 2 (2.0) | 34 (34.0) | 1 (1.0) |
| Exercises | **89 (89.0)** | 0 (0.0) | 55 (55.5) | 1 (1.0) | **86 (86.0)** | 0 (0.0) |
| Advice on stress management | 39 (39.0) | 0 (0.0) | 51 (51.5) | 1 (1.0) | 32 (32.0) | 0 (0.0) |
| Advice on food | 16 (16.0) | 0 (0.0) | 26 (26.3) | 1 (1.0) | 12 (12.0) | 0 (0.0) |
| Advice on active living | **79 (80.6)** | 2 (2.0) | 60 (60.6) | 1 (1.0) | 69 (69.0) | 0 (0.0) |
| Advice on headache triggers | 60 (60.6) | 1 (1.0) | 63 (64.3) | 2 (2.0) | 62 (63.3) | 2 (2.0) |
| Electrotherapy | 1 (1.0) | 3 (3.0) | 4 (4.2) | 5 (5.0) | 4 (4.0) | 1 (1.0) |
| Other* | 6 (9.5) | 37 (37.0) | 3 (4.7) | 36 (36.0) | 4 (6.7) | 40 (40.0) |

* Answer options: Always, Often, Sometimes, Rarely, Never. This table includes only respondents who selected ‘Always’ or ‘Often’

**Other: Ergonomics, Advice on analgesics, Advice on sleep, Cryotherapy, Patient education and Integration of primary reflexes.

Bold: modalities used by more than 75% of the chiropractors

Table S3 Chiropractors’ self-rated use of monitoring tools in patients with headache

|  | Total N | Never | Rarely | Sometimes | Often | Always |
| --- | --- | --- | --- | --- | --- | --- |
| Headache diary, n (%) | 100 | 33 (33.0) | 30 (30.0) | 30 (30.0) | 6 (6.0) | 1 (1.0) |
| Headache calendar, n (%) | 100 | 37 (37.0) | 31 (31.0) | 27 (27.0) | 4 (4.0) | 1 (1.0) |
| Other, n (%)* | 79 | 65 (82.3) | 3 (3.8) | 2 (2.5) | 3 (3.8) | 6 (7.6) |

* An app “My headache”, asking about the patient’s own experience, food and fluid tracing.

Table S4 Most common side effects noted by the chiropractors

| Side effects noted* | n (%) |
| --- | --- |
| Provocation of known headache / symptoms | 57 (38.0) |
| Soreness | 38 (25.3) |
| Tiredness | 30 (20.0) |
| Dizziness | 10 (6.7) |
| Nausea | 8 (5.3) |
| Uncomfortable / "hangover like" | 5 (3.3) |
| Tearful | 1 (0.7) |
| Thirsty | 1 (0.7) |

 * Multiple answers possible
