# Supplementary material for: Diagnostic approach and management of patients with headache in Danish chiropractic practice
Source: Chiropr Man Therap. 2026 May 29;34:29. doi: 10.1186/s12998-026-00652-0 (PMC13419007; doi:10.1186/s12998-026-00652-0)
Supplement: Supplementary file 3 — Supplementary Material 3 [file 12998_2026_652_MOESM3_ESM.docx]

## Additional File 1: Supplemental Figures

Figure S1 Chiropractors’ self-rated content of medical history and physical examination according to the Danish profession-specific clinical care standard


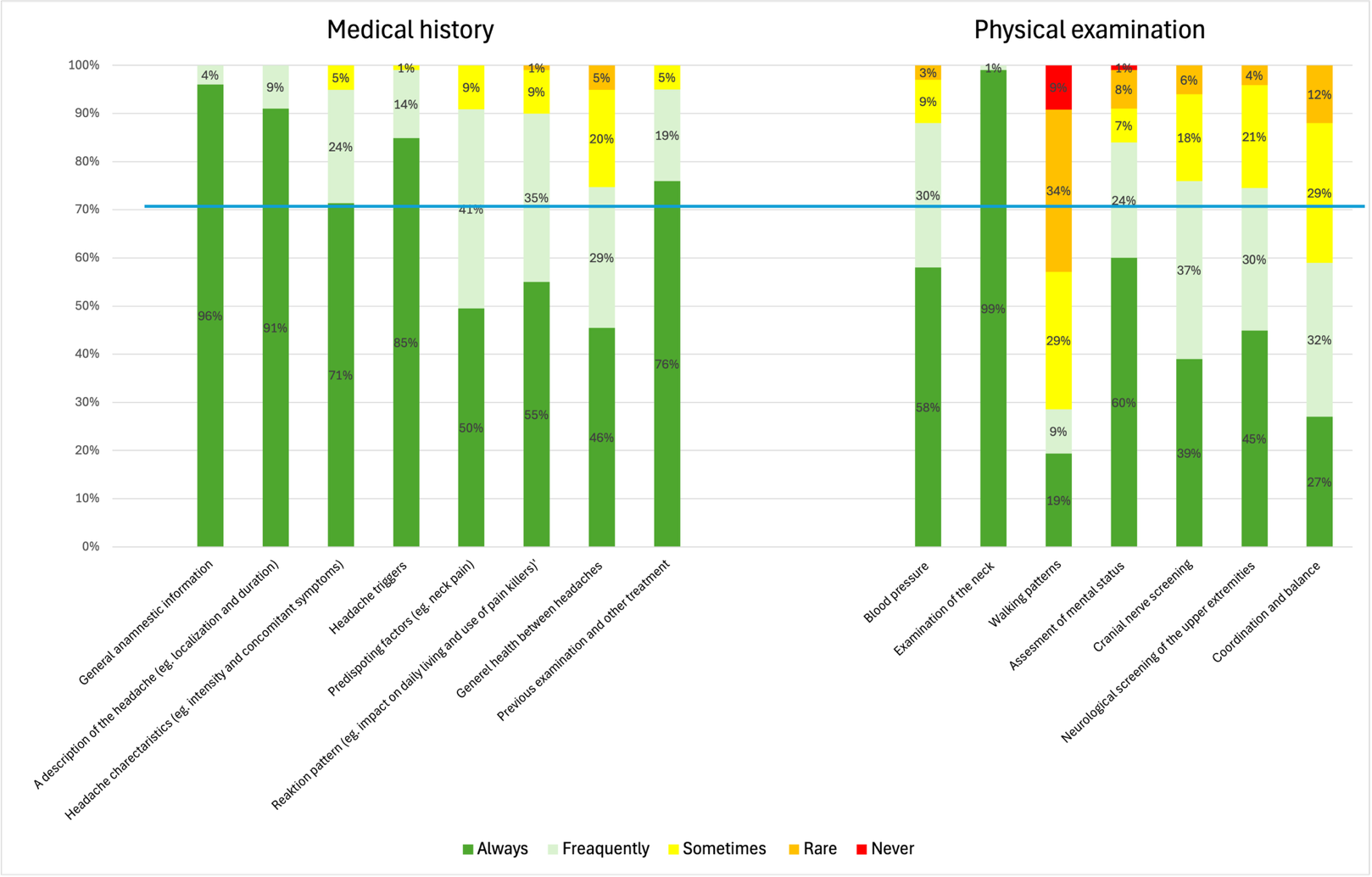


Blue line: 70% threshold for adherence

Figure S2 Chiropractors’ self-rated use of x-ray in the diagnosis and management of headaches
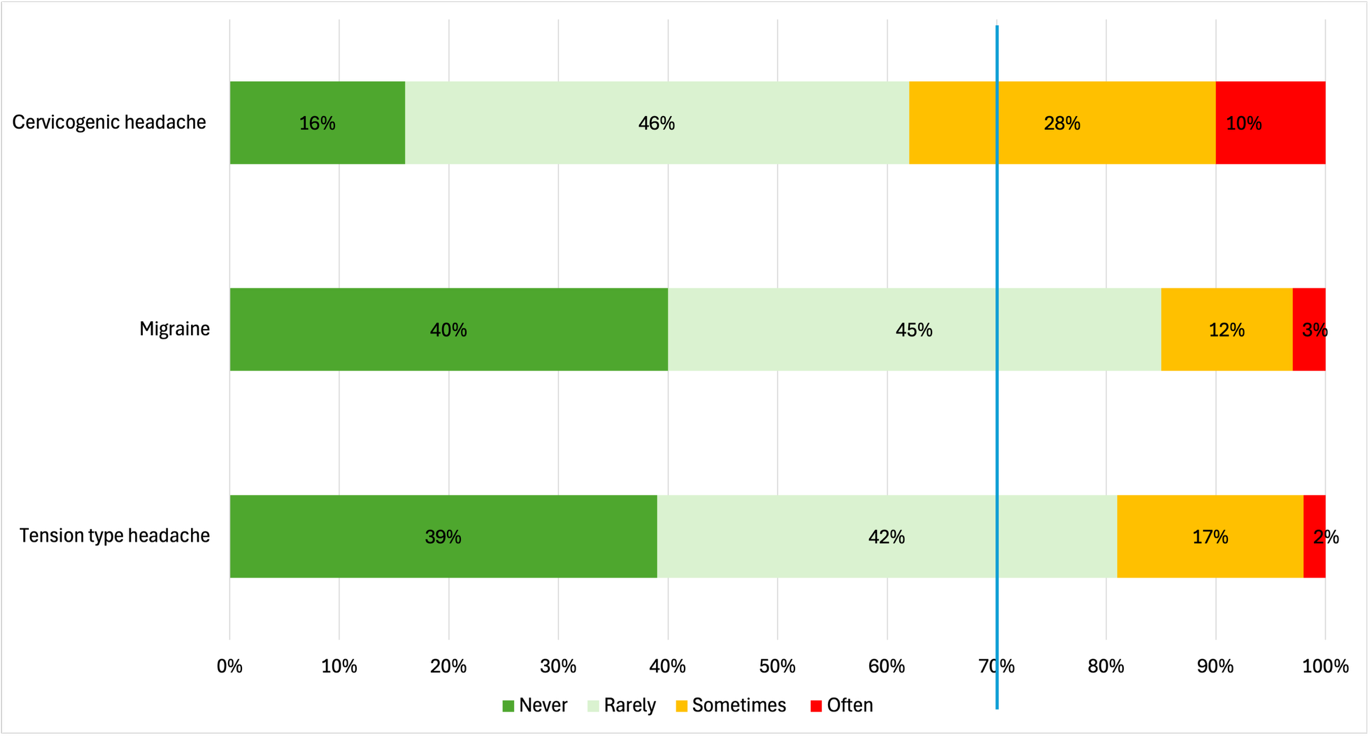


Blue line: 70% threshold for adherence

Figure S3 Chiropractors’ self-rated knowledge of red flags
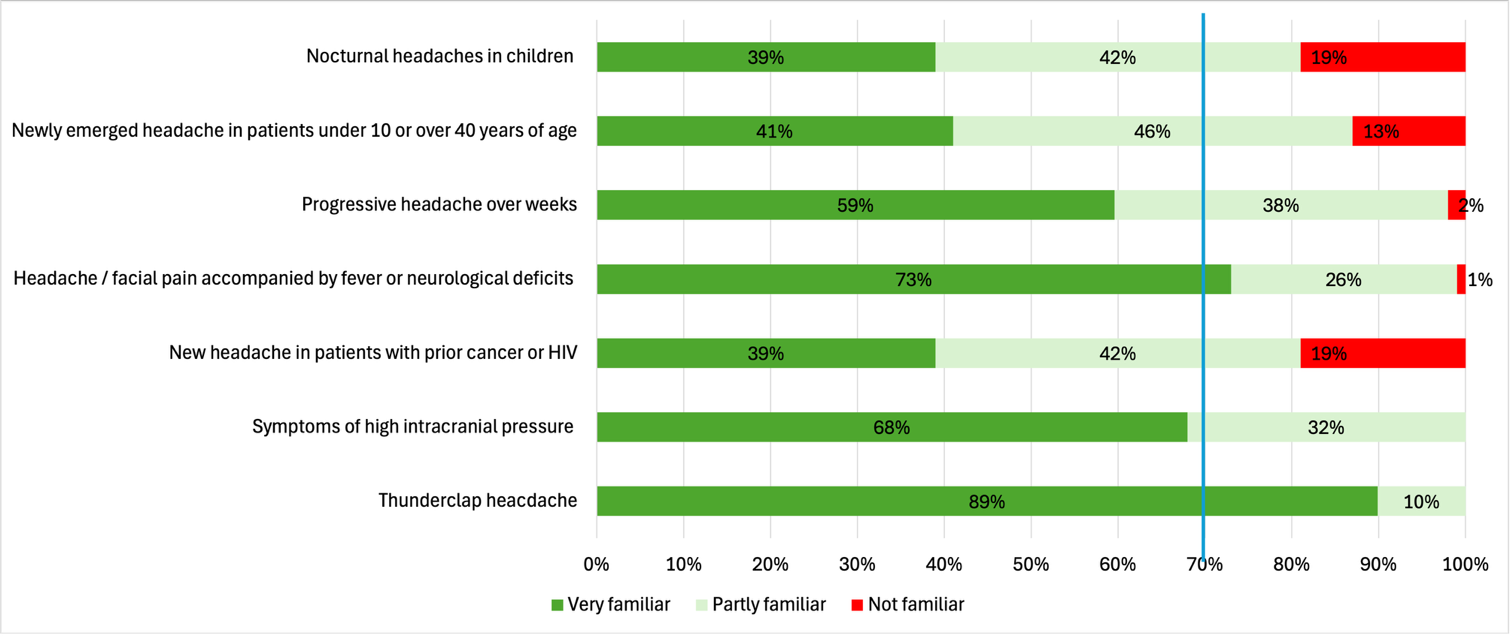


Blue line: 70% threshold for adherence

Figure S4 Chiropractors’ attitude towards diagnostic criteria for primary headaches regarding the usage in a clinical setting


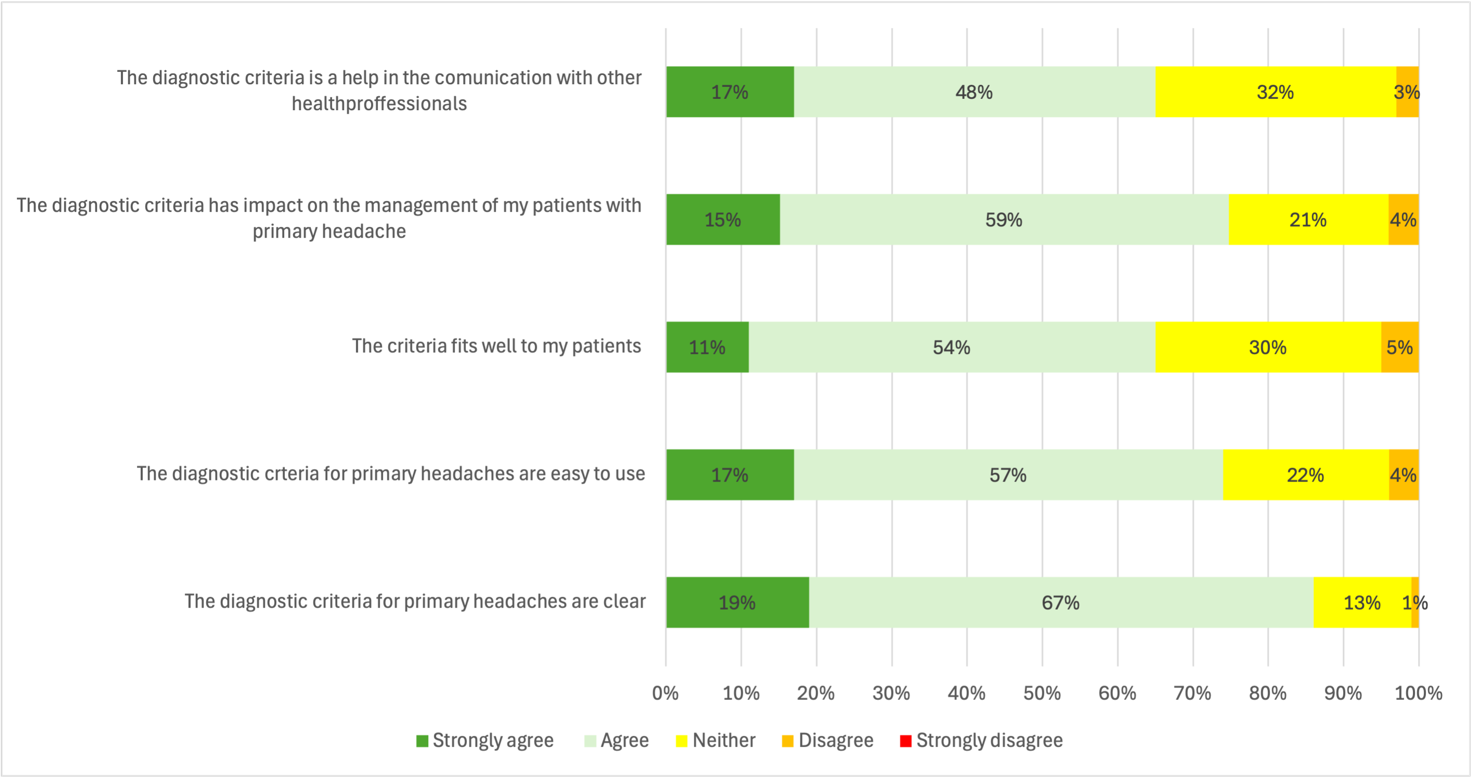


Figure S5 Chiropractors’ attitude towards diagnostic criteria for secondary headaches regarding the usage in a clinical setting
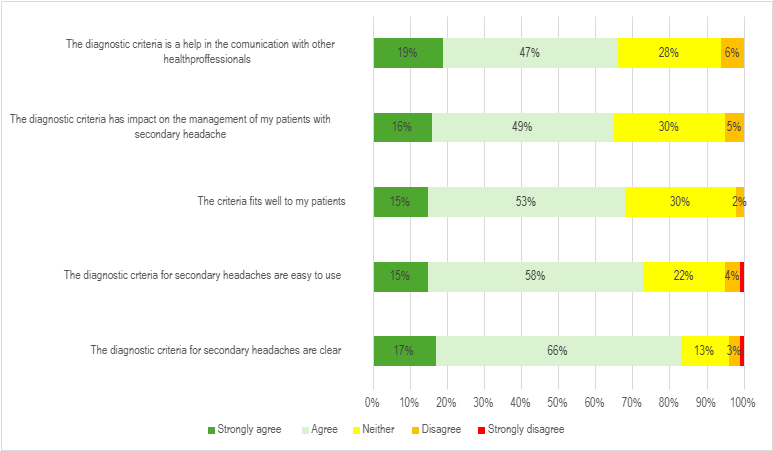


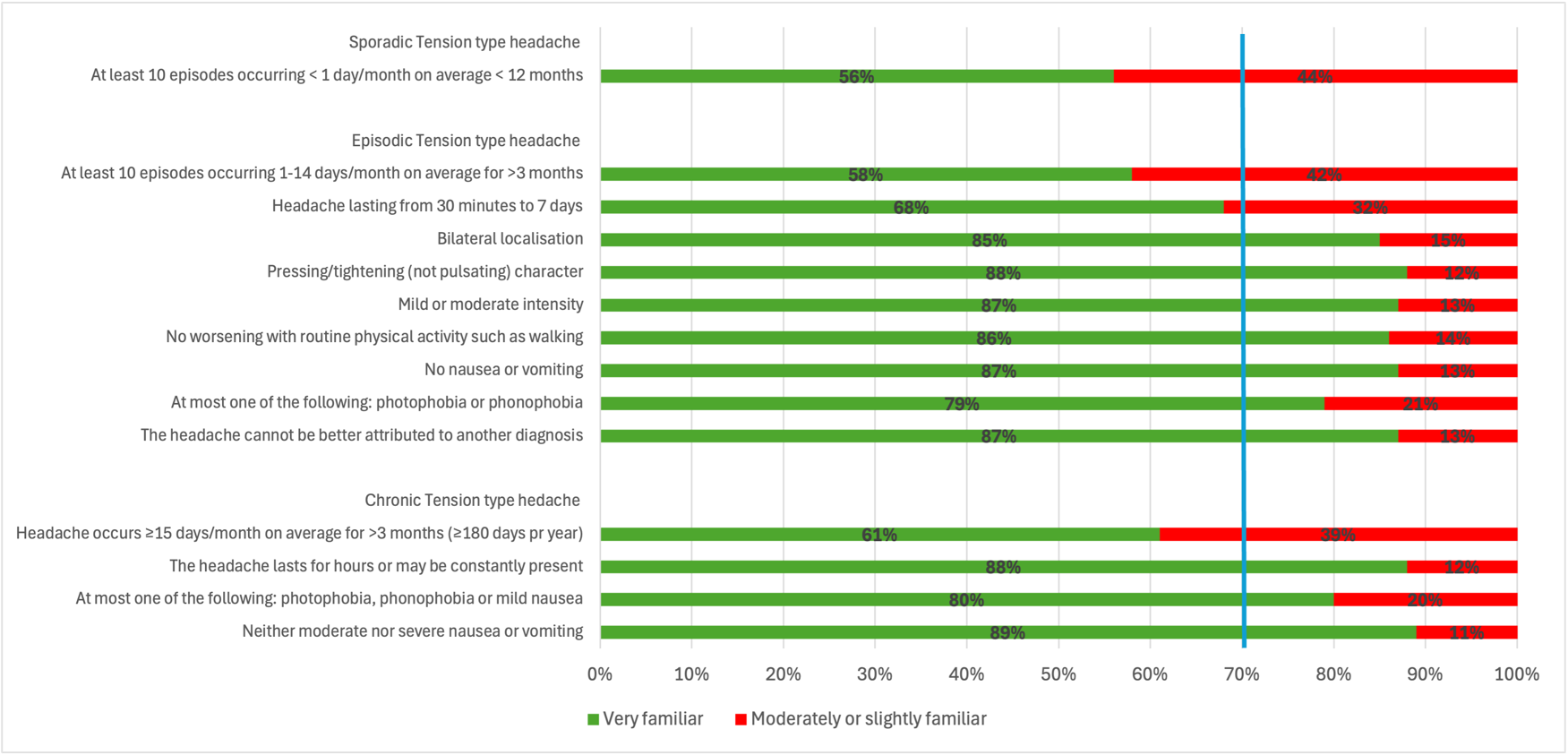
Figure S6 Self-rated knowledge of tension-type headache (sporadic, episodic and chronic)

Blue line: 70% threshold for adherence

Figure S7 Self-rated knowledge of cervicogenic headache


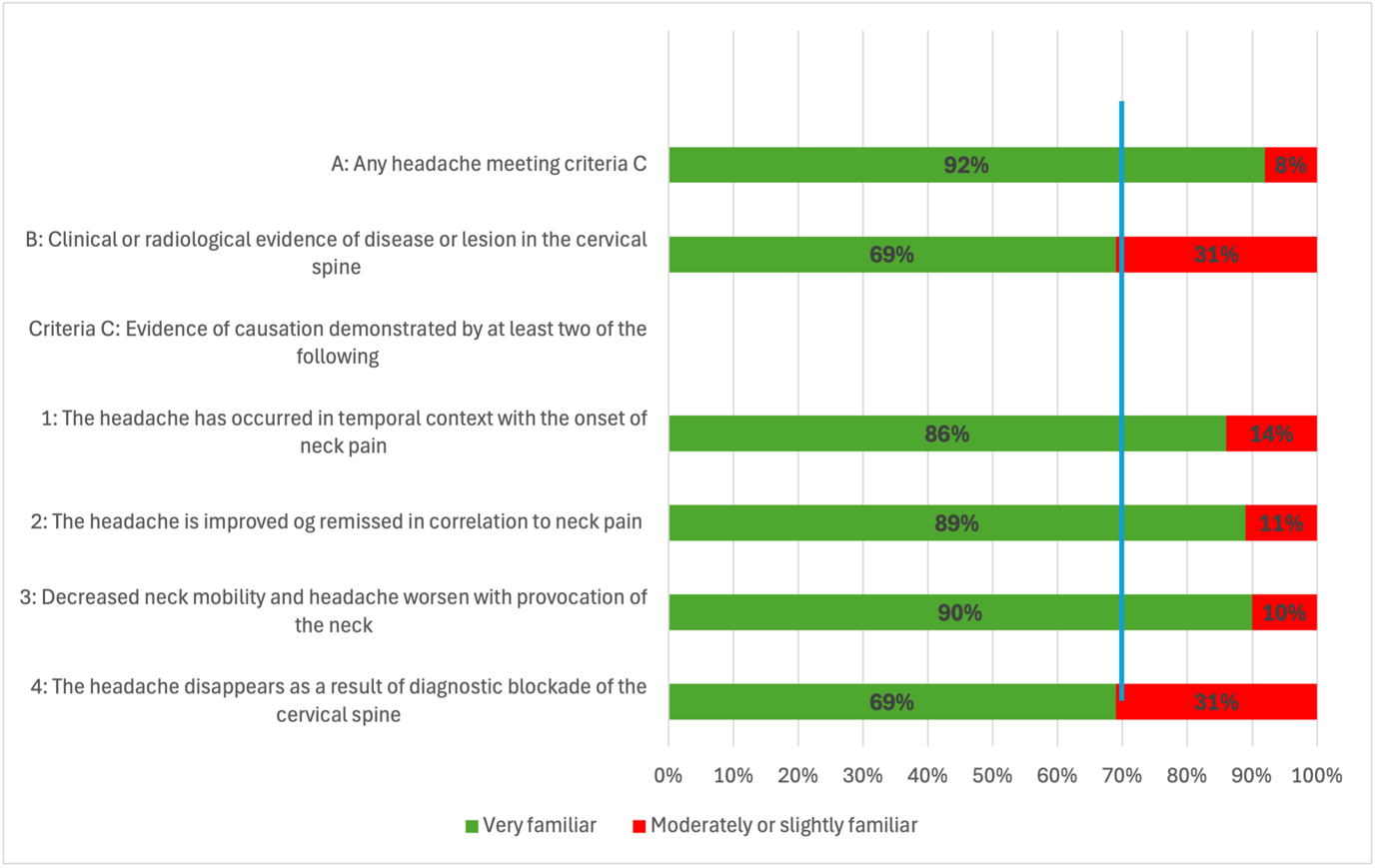


Blue line: 70% threshold for adherence

Figure S8 Self-rated knowledge of migraine


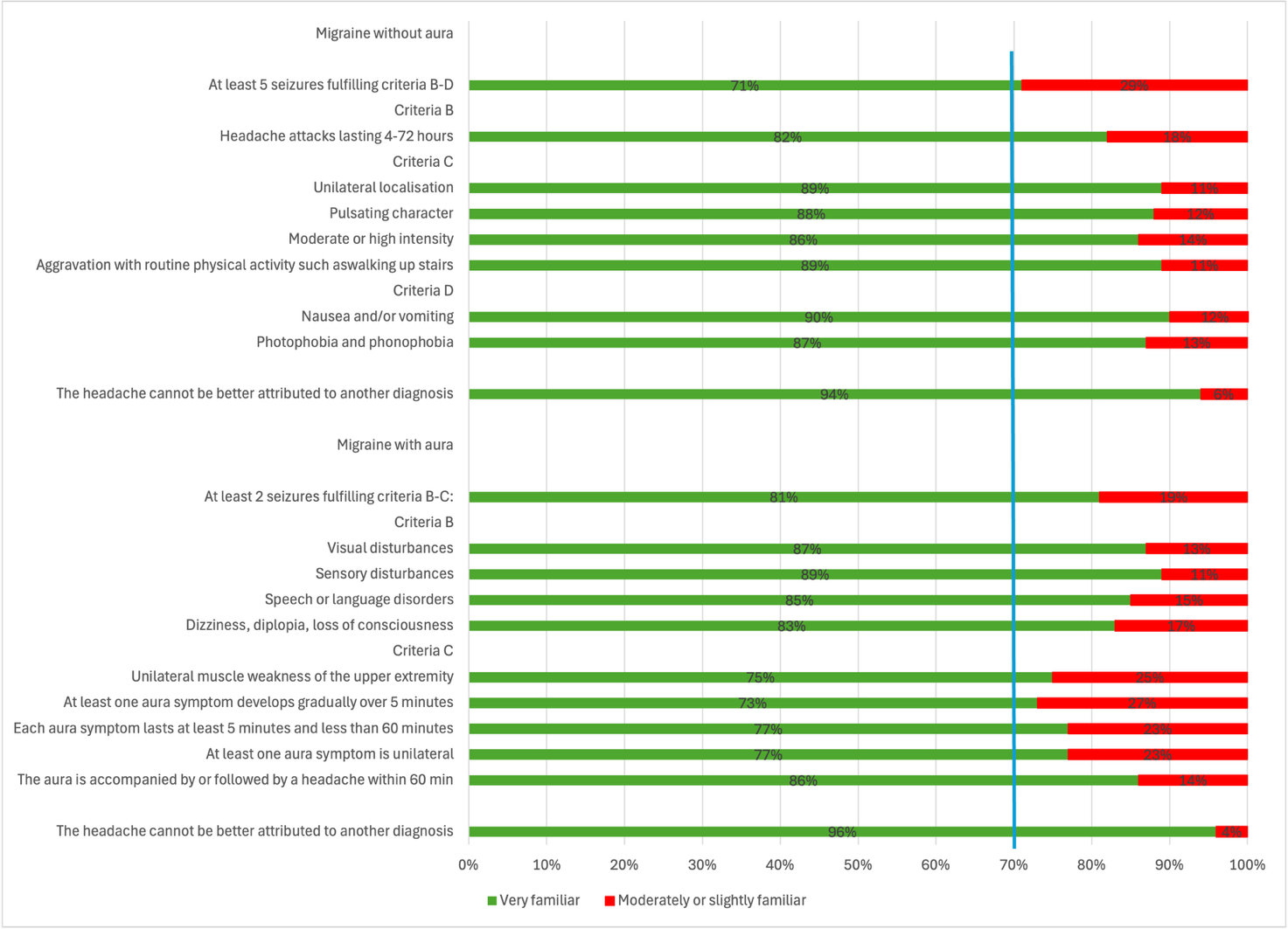


Blue line: 70% threshold for adherence
